# Supplementary material for: Chromothripsis during telomere crisis is independent of NHEJ, and consistent with a replicative origin
Source: Genome Res. 2019 May;29(5):737–49. doi: 10.1101/gr.240705.118 (PMC6499312; doi:10.1101/gr.240705.118)
Supplement: Supplemental Material [file supp_gr.240705.118_Supplemental_file_1.zip › contigs/annotated_contigs/DB75/contig.2.DB75_length_647_mean_cov_8.114374034.docx]

**DB75_length_647_mean_cov_8.114374034**

```
TGGGCAAGTTTATCCTATTGATTTTGAAAGAAATACTTAGAAAAATATTGCTTCTACCAATGCTATTTTTCAGGTTTGTGCTGATAGTG
 >chr3:54374126-54374306 + E=1e-96
TTCCCTACTCACAACATTTTATTATGAAAATATCCAGACATGCAACACAATTGAAACAAATTTGCCACACAAACACCCATACACCCCCC

AT|ACA|CCCCTTGTCCCCCTCGCAGGGCGTGCGATGGGGGTGTGGCTCGCTTCTTCACTGCCTTGTTGCTCAAACCTCTAGGGGAGCA
      >chr3:54237268-54237732 + E=1e-264
TACAGATGGGCAGGCTGTGGGGCTCTGACTCCATGGCGGTGTCTAGGGGTGAATGTTTACAGCTCCTGAAGCCCTTGTGGGTGTGTGTT

ACAGGGTGCTCCTTTAGTTTGTCATCTGTAGGCAGCTTGTGTTAGCTCAATTAGACCCTCTACCTTGTTGCAAGGACAAAGGGCTTTCT

GTATCCCGGGTTCTTGCCTTGGTATACTGGAAGAATCTGATCACACATGAGCTTGGAAAATGAGGGCAAAGTTTTATTGAGTGGAAGCA

GCTCTCCGCTGATGGGGAAGCCAGAAGGGAGATGGTTTTCCTCTGGAGTTGGGCCACTGTGTGGCCCCAGTTCTCCTTCAACTCCCTCG

GCCAAACTCCGTCTCATCCCGCTGGT
```
